# Non-Invasive Forehead Segmentation in Thermographic Imaging

**DOI:** 10.3390/s19194096

**Published:** 2019-09-22

**Authors:** Francisco J. Rodriguez-Lozano, Fernando León-García, M. Ruiz de Adana, Jose M. Palomares, J. Olivares

**Affiliations:** 1 Department of Electronic and Computer Engineering, Universidad de Córdoba, Edificio Leonardo da Vinci, Campus de Rabanales, 14071 Córdoba, Spain; fernando.leon@uco.es (F.L.-G.); jmpalomares@uco.es (J.M.P.); olivares@uco.es (J.O.); 2 Department of Chemical, Physics and Applied Thermodynamics, Universidad de Córdoba, Edificio Leonardo da Vinci, Campus de Rabanales, 14071 Córdoba, Spain; manuel.ruiz@uco.es

**Keywords:** image processing, forehead segmentation, thermographic imaging, computer vision, body parameters

## Abstract

The temperature of the forehead is known to be highly correlated with the internal body temperature. This area is widely used in thermal comfort systems, lie-detection systems, etc. However, there is a lack of tools to achieve the segmentation of the forehead using thermographic images and non-intrusive methods. In fact, this is usually segmented manually. This work proposes a simple and novel method to segment the forehead region and to extract the average temperature from this area solving this lack of non-user interaction tools. Our method is invariant to the position of the face, and other different morphologies even with the presence of external objects. The results provide an accuracy of 90% compared to the manual segmentation using the coefficient of Jaccard as a metric of similitude. Moreover, due to the simplicity of the proposed method, it can work with real-time constraints at 83 frames per second in embedded systems with low computational resources. Finally, a new dataset of thermal face images is presented, which includes some features which are difficult to find in other sets, such as glasses, beards, moustaches, breathing masks, and different neck rotations and flexions.

## 1. Introduction

In recent decades, the main mechanism used to obtain the temperature of a human body was the usage of some invasive devices or which were placed in contact with the skin. For instance, thermometers located in specific parts of the body, such as the forehead, the rectum, or the armpit. These traditional practices have proved to work well. However, in some cases, it is not possible to use contact-based or invasive devices due to hygienic issues. For instance, when it is necessary to take the temperature of many people in a short time [1]. Another example in which they are not suitable is when the temperature of a person varies rapidly with different emotions [2].

Current technology in thermal images [3,4] allows the creation of less invasive systems which avoid wires and use contactless sensors. In this scenario, thermography has evolved as a clear advance in the remote temperature sensing area [5,6]. These advantages are useful for obtaining the temperature of a body without invasive methods. Moreover, thermography allows the acquisition of temperature in specific zones of the body, such as the forehead.

The skin forehead temperature is correlated with the body core temperature [7] because of the large vascularization and thin skin in that zone. This correlation means that when a person suffers from hyperthermia, the temperature of the forehead is higher than usual. On the other hand, if the person is suffering from hypothermia, the forehead shows a lower temperature. This behavior is not consistent in other parts of the human body, for instance, in the limbs, where temperature may vary largely from the body core temperature. This behavior has made the forehead to be widely used with high relevance in different works. These works cover from fever detection systems [1], healthcare systems [8], climatic comfort systems [9] to psychophysiology measurement systems [10], among others.

All these previously mentioned proposals have two points in common. The first one, the forehead region has been used as a key indicator for all the different scenarios. This selection of the forehead shows the importance and relevance of that region for many different applications. The second point in common for all those systems is the acquisition method of the temperature of the forehead area. That acquisition has been usually carried out manually. Currently, there are few tools to acquire this area using an automatic approach with real-time constraints in thermal images.

Hence, the purpose of this work is to obtain the region of the forehead in thermal images and acquire the average temperature of the forehead using a single thermal camera in a non-invasive way. The system needs to be invariant in two ways: it should be able to handle movements, mainly rotations of the head, and it should manage different morphologies of the human head (people with different facial features, longer or shorter noses, glasses, beards, etc.). The proposed system works with all of them and only requires user interaction to highlight when the person in front of the camera is wearing glasses. Another contribution of this work is a dataset over of 18000 thermal images including the aforementioned features.

Since the current work aims to automatically obtain the forehead region, and the average temperature from that region, this work has a relation with face detection algorithms. Face detection and classifications problems [11,12,13,14] had been widely analyzed in the past and seem to be solved. However, the acquisition of the forehead region and fluctuation temperature using thermal sensors with real-time constraints is not available using the current set of tools for face detection and identification.

As a summary, in this work, we propose an automatic method to detect the forehead area and to extract the average temperature of the forehead using images provided by a single thermal camera. This paper is organized as follows: a brief study of the current works that use the forehead region is presented in Section 2. In Section 3, the proposed method is described. The materials used and the followed procedure to carry out the experiments are shown in Section 4. The results are analyzed and discussed in Section 5 and Section 6, respectively. Finally, the main conclusions are presented in Section 7.

## 2. Related Works

In this section, several proposals with different aims are analyzed. However, a point of interest in common is clear, all of them use the forehead area or the forehead temperature as an important part in their research.

Fever is a concept usually linked to the forehead region and this relation is described in the work of Somboonkaew et al. [15]. In their work, an automatic method to extract the highest temperature of the face and the forehead region using mobile devices is proposed. The proposal of these authors uses an RGB camera with which they perform the detection and acquisition of the face and forehead, using the thermal camera only to extract temperature. However, their proposal is based on an RGB face detection algorithm which cannot be applied directly to thermal images. This problem is intensified when the user has different facial elements that modify the morphology of the face.

Another example of using an RGB camera and the forehead region is detailed in the work of Kerr et al. [8]. Their work demonstrated how texture based on colors is a marker for the health condition of a person. Their work focused on analyzing the Capillary Refill Time (CRT). The texture of the forehead region and the time that the skin takes to recover its color were used. A robot hand was used to make the pressure on the skin of the forehead and to acquire the images of that zone. However, in their work, the forehead region was manually segmented.

A different topic is studied in the work of Koukiou [16], where a system was proposed to detect when a person is intoxicated by alcohol. Several points and regions of the face were used, including the forehead. The temperature of the forehead was used as an important indicator to know when a person is intoxicated by alcohol. Their work concludes that the forehead region provides 90% of success to detect intoxicated persons. However, the extraction of the forehead was done manually.

The forehead region has been used not only to detect intoxicated persons, but in other proposals with a psychophysiology focus. An example of this is the work of Ioannou et al. [10]. The authors describe the system, in which several different points such as the forehead, periorbitals, nose, cheeks, and some others were acquired manually from thermal images. The selected points were analyzed and tabulated giving valuable information about the variation of temperature depending on their emotions. Their work showed that the forehead is especially sensitive to stress, sexual arousal, and anxiety emotions.

Zhu et al. [17] use the forehead region to analyze when a person is under stress, instead of the eye areas, as it is usually used in other works. From the forehead region, the authors obtained the thermal signature of the corrugator muscle. This muscle was analyzed, ensuring it varies in shape and temperature when a person is under stress or when they are lying. This tool for lie detection presents a 76.3% of successful detected cases using the forehead region. However, the corrugator muscle and the forehead region were segmented manually.

Psychophysiology measurements are used with other systems to improve them. For instance, the system named StressCam [2] was proposed for analyzing the frustration of the users using Human-Computer Interaction (HCI). The authors selected manually a region of interest of the forehead. Once the forehead region was selected, a tracking algorithm was used to track the movements of the face. 10% of the hottest spots inside the forehead region were used to quantify the frustration emotion. This detection is based on the evolution of the temperatures, which are directly correlated with the blood flow activity. Even though the authors monitored the forehead region, that area was manually selected at least once, at the initial stage.

However, the temperature of the forehead region is not only limited to social measurement indicators. Thermography has been used to analyze thermal comfort. Oliveira et al. [9] use the forehead region, the cheeks, the periorbital of the eyes, and the nasal temperature to determine thermal comfort of the users. The authors consider that significant differences exist in the temperature of the right and the left side of the region of the forehead. The authors confirmed experimentally that there is clear evidence of a correlation between the thermal comfort and the areas chosen for the analysis. The areas of interest, such as the forehead, were manually selected.

Another example of the comfort estimation using the forehead area was developed by Ghahramani et al. [18]. The authors proposed a system which collects the temperatures using glasses and small thermal sensors. This system is intended to improve the Heating, Ventilation, and Air Conditioning (HVAC) systems based on thermostats. The system does not need user intervention and monitoring. The authors were able to confirm experimentally with 95% of confidence that the forehead region and other points of the face are indicators of the thermal comfort. The main disadvantage is that the authors proposed a contact-based system which does not allow the freedom of movements of users. Moreover, if used for studies similar to the aforementioned ones, many false positives may arise, especially in those people who do not usually wear glasses.

Lubkowska et al. [19] conducted a study using thermographic images to analyze the temperature of various body parts of newborns during their first minute of life. One of the areas that have relevance in their study is the forehead. The authors worked with 74 recordings of newborns. In all these recordings, the forehead area was manually segmented. Therefore, the process was carried out offline without any real-time constraint.

Bando et al. [20] conducted a study to analyze the temperature variations in the forehead area in people with induced drowsiness. Their study was conducted with seven men and women for whom the forehead area was manually selected to carry out the study. After analyzing the different states and evolution of forehead temperature, the authors concluded that the forehead area may be a key factor in detecting drowsiness in its most premature states. As detailed by the authors, this fact can be applied in vehicles to reduce the number of accidents caused by drowsiness. This work, as the proposal of Lubkowska et al. [19], would benefit from an automatic, non-invasive forehead segmentation system that works with real-time constraints.

There is some work focused on extracting features from the face in thermography images. Marzec et al. [21] proposed a method to extract the features (such as eyes, periorbitals, eyebrows, forehead, nose and so on) of the face using thermal images. To acquire these features, the head of a person is segmented from the background of the image using thresholding. After that, they extract the center of the head and fit it in a curve. After normalization of the position of the forehead, the features are extracted based on the morphology of the head. However, although this work presents some similarity to the objectives pursued and methods used in our proposal, the work of Marzec et al. is strongly linked to morphology and cannot be used in general scenarios when a user wears glasses or other elements that hide or modify the morphology of the shape of the face.

A similar work to the previously cited one is proposed by Trujillo et al. [22]. In that, the facial features of a face in thermal images were segmented. These features are used to recognize the facial expression of a person. They extract the mouth, the eyes, and the nose. Those features are used in a classification model based on a Support Vector Machine Committee approach. In this case, the forehead area is not used, but their work has some points in common with our work, as the detection of the eyes and the glasses. However, that proposal, and the proposal of Marzec et al. [21] cannot be used when different objects or even the beard of a person is in the face, since their proposal finds some points based on the shape of an ideal face.

In addition to the aforementioned works, Robinson et al. [23] remark in their work that there are difficulties in analyzing a thermal image. There are elements such as glasses, hair, masks, which hinder the acquisition of temperatures. These elements represent a challenge when analyzing thermography images. These elements are covered in our proposal.

Once the related works have been analyzed, it can be deduced that most of the related works using the forehead region use a manual segmentation approach. On the other hand, in those works where the forehead or features of the face are automatically acquired, the proposals are highly linked to the morphology of the face and its relation to an ideal standard-shaped face. Hence, an automatic approach is necessary to boost the potential of these proposals and future works based on forehead temperature acquisition.

## 3. Methodology

The proposed method to solve the segmentation and extraction of the forehead temperature is shown in Algorithm 1.

**Algorithm 1:** Steps of forehead temperature extraction** 1**  **PROPOSED_METHOD** (*raw_frame*, *hasGlasses*); ** 2**  *grayScale_frame* ← Raw_data_transformation (*raw_frame*); ** 3**  *thresholded_frame*, *binarized_frame* ← threshoding_image (*grayScale_frame*); ** 4**  *ellipse* ← ellipse_computation (*binarized_frame*); ** 5**  *upper half_ellipse* ← extraction_upper-half_ellipse (*ellipse*); ** 6**  **if**
*hasGlasses is FALSE*
**then** ** 7**    |   *eyes_glasses* ← eyes_detection (*upper half_ellipse*, *thresholded_frame*); ** 8**  **else** ** 9**    |   *eyes_glasses* ← glasses_detection (*upper half_ellipse*, *thresholded_frame*); **10**  **end** **11**  *forehead_mask* ← forehead_segmentation (*eyes_glasses*); **12**  *forehead_temperature* ← forehead_temperature_extraction (*raw_frame*, *forehead_mask*); **13**  **Return** forehead_temperature;


Algorithm 1 takes as input a *raw_frame* obtained from a single thermal camera and processes it to obtain the temperature of the forehead region. Moreover, depending on whether a person wears glasses or not, the system has a slightly different behavior (*hasGlasses* Boolean variable). The following subsections explain the method step by step.

### 3.1. Raw Data Transformation

**Figure 1:**
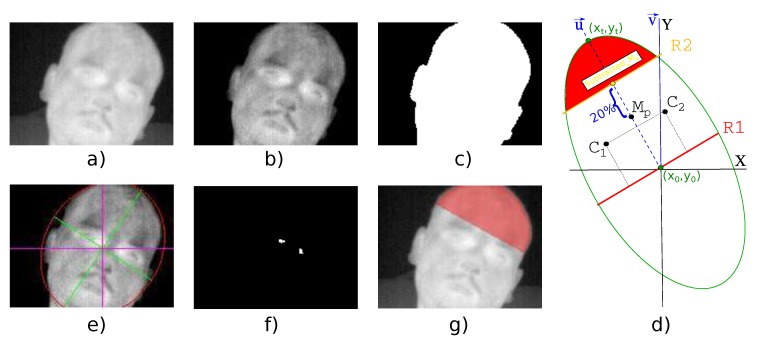
Different steps of the proposed method. (**a**) Grayscale image; (**b**) Thresholded image; (**c**) Binary mask; (**d**) Vector directors and main points to extract forehead region; (**e**) Ellipse calculation; (**f**) Eyes detection; (**g**) Forehead region overlapped with grayscale image.

The first step of the proposed method, as Algorithm 1 details, is a transformation of the raw thermal data (temperature matrix in the range [−20 °C–100 °C]) obtained from the used sensor (detailed in Section 4) into an 8-bit grayscale. 8-bit grayscale images represent all pixels in the range [0–255], which 0 value is the lowest temperature value. This transformation simplifies the step detailed in Section 3.2 since only integer values in a positive range are considered. The conversion of raw frames into grayscale images is performed by Equation (1).

(1) $$P^{\prime }\;\left(x,y\right)=\left(\frac{P\;(x,y)-min}{max-min}\right)\times 255$$

where P(x,y) is a point from thermal image, $P^{\prime }\;\left(x,y\right)$ is a value in grayscale. The pair min and max represents the lowest and highest value in the raw frame. The result of this step is shown in Figure 1a.

### 3.2. Threshoding Images

The purpose of this step is to remove those pixels from the grayscale image which belong to the background. This is performed with a linear transformation of each pixel in the grayscale image. This transformation is performed using Equation (2). The visual result is shown in Figure 1b.

(2) $$P^{\prime \prime }\;\left(x,y\right)=\left\{\begin{array}{ccc} 0 & ,\mathrm{if} & P^{\prime }\;\left(x,y\right)\leq M\\ 255\times \frac{P^{\prime }\;\left(x,y\right)-M}{255-M} & ,\mathrm{if} & M<P^{\prime }\;\left(x,y\right)\leq 255 \end{array}\right.$$

where $P^{\prime }\;\left(x,y\right)$ is a point in grayscale obtained by Equation (1) and $P^{\prime \prime }\;\left(x,y\right)$ is a new value for each point of the image. Finally, *M* is the used parameter to transform each pixel.

The value of *M* can be calculated using the Otsu method [24]. Otsu method splits a grayscale image into two sets $S_{0}=\left[0,M\right]$ and $S_{1}=\left[M+1,255\right]$, where *M* is used as a threshold in order to minimize the weighted within-classes variance. Otsu method is computed using Equation (3). Where $\sigma_{M}^{2}$ is the variance and $P_{i}$ is the probability of each grayscale value.

(3) $$\sigma_{M}^{2}=\sum_{i=0}^{M}P_{i}\times \sum_{i=M+1}^{255}P_{i}\times {\left(\frac{\sum_{i=0}^{M}iP_{i}}{\sum_{i=0}^{255}P_{i}}-\frac{\sum_{i=M+1}^{255}iP_{i}}{1-\sum_{i=0}^{255}P_{i}}\right)}^{2}$$

To simplify the following steps of the method, a binary mask is used to represent the head with non-zero-valued pixels. This task is carried out using Equation (4).

(4) $$P^{\prime \prime \prime }\;\left(x,y\right)=\left\{\begin{array}{ccc} 0 & ,\mathrm{if} & P^{\prime \prime }\;\left(x,y\right)=0\\ 255 & ,\mathrm{if} & 0<P^{\prime \prime }\;\left(x,y\right) \end{array}\right.$$

where $P^{\prime \prime }\;\left(x,y\right)$ is a point of the thresholding image, and $P^{\prime \prime \prime }\;\left(x,y\right)$ is a point of the binary mask. An example of a generated binary mask is shown in Figure 1c.

Using the binary mask, an erosion morphology operator [25] of that mask followed by an *XOR* logic operator is calculated. The erosion operator contracts the binary image and the *XOR* logic operator applied to the binary mask allows for the detection of the boundaries of the binary image.

### 3.3. Ellipse Computation

The purpose of this section is to fit the set of points generated in the previous step to an ellipse. In general terms, the ellipse is the geometric figure that best fits the head of a user. This step uses the method proposed by [26] to perform a robust ellipse fitting.

The equation of conic sections (including ellipses and some other shapes) is represented by Equation (5).

(5) $$F\left(x,y\right)=A\times x^{2}+B\times xy+C\times y^{2}+D\times x+E\times y+F=0$$

where (x,y) is the coordinate of a point, which belongs to the ellipse, and A,B,C,D,E,F are the parameters of the ellipse equation. The sets of parameters that correspond with an ellipse are defined by $B^{2}-4AC=1$ as the work in [26] details.

Hence, the ellipse fitting problem can be solved by minimizing the sum of least squares as detailed in Equation (6).

(6) $$\min_{\theta }\sum_{i=1}^{n}{\left(\theta \times \omega \right)}^{2}=\min_{\theta }\sum_{i=1}^{n}{\left(A\times x_{i}^{2}+B\times x_{i}y_{i}+C\times y_{i}^{2}+D\times x_{i}+E\times y_{i}+F\right)}^{2}$$

where *i* represents each point of the set point to fit an ellipse, and *n*, the number of available points. $\theta ={A,B,C,D,E,F}^{T}$ is a vector composed by the parameters of the ellipse equation and $\omega =x^{2},xy,y^{2},y$ is a vector composed by the variables of Equation (5).

This approach has been used instead of the Generalized Hough Transform [27] because this last one is computationally more complex and has a larger computation time than using the simple method to fit the head to an ellipse shape. The result of the ellipse fitting step is shown in Figure 1e.

### 3.4. Extraction Upper Half Ellipse

Once the ellipse has been found, it is cut to simplify the following steps, since the forehead is a region placed in the upper half of the ellipse. For this reason, the method will remove all the points that are below the minor axis of the ellipse. The minor axis of the ellipse corresponds to a line ($R_{1}$) through the central point of the ellipse ($x_{0}$, $y_{0}$) and the point where the semi-axis cuts the ellipse ($x_{l}$, $y_{l}$). Since there are two points that cut the ellipse, only one of them it is necessary. In this case, the left point is chosen (for the right point it would be done in a similar way). Equations (7) and (8) show how to perform the calculation of the center point of the ellipse and the left point, respectively.

(7) $$x_{0}=\frac{B\times E-2\times C\times D}{4\times A\times C-B^{2}}\;y_{0}=\frac{B\times D-2\times A\times E}{4\times A\times C-B^{2}}$$

(8) $$x_{l}=y_{0}+\frac{\sqrt{{\left(2\times B\times E-4\times C\times D\right)}^{2}+4\times \left(4\times A\times C-B^{2}\right)\times \left(E^{2}-4\times C\times F\right)}}{2\times (4\times A\times C-B^{2})}\;y_{l}=\frac{-B\times x_{l}-E}{2\times C}$$

The line representation is defined by Equation (9), where *m* is the slope of the line and *b* the point where the line crosses the ordinate axis:

(9) $$y=m\times x+b\;m=\frac{y_{l}-y_{0}}{x_{l}-x_{0}}\;b=y_{0}-\left(m\times x_{0}\right)$$

Once the line is found, a point $P_{p}\left(x_{p},y_{p}\right)$ is in the upper half of the ellipse, if it satisfies the condition $y_{p}-m\times x_{p}+b<0$. In that case, this point is set to 0 in the mask. Hence, in the following steps, only the upper half of the ellipse is used.

### 3.5. Eyes/glasses Detection

Eyes are located close to the forehead. Hence, eyes are a desirable region to detect the forehead area. The location of the eyes can be obtained using the K-means algorithm [28], because eyes have a higher temperature [29] than the rest of temperature values in the upper-half of the ellipse. An example of eye temperature is shown in Figure 1f.

This method can detect the position of the eyes, unless an object covers the thermal radiation emission of the eyes. For instance, lenses of the glasses prevent the acquisition of thermal temperature emitted by eyes and the surrounding area. In these cases, a slightly different method has been designed to deal with users wearing glasses since the thermal radiation emission of this zone is different.

In general terms, this step works in the same way for users with or without glasses. For users without glasses, K-means will search for clusters with the highest temperature inside the face. On the other hand, for users with glasses, K-means will search for clusters of points with the lowest temperature inside the face. The materials typically used for lenses are polycarbonate, mineral glass, and organic material. These elements provide different emission values compared to the face. Hence, their temperature tends to be always different (mostly lower) than the rest of the face.

In this step, the system must know whether the user wears glasses or not, to select the correct approach of K-means. This is obtained by user selection and is the only parameter required from the user.

Nevertheless, K-means is sensitive to the number of clusters and the position of the initial clusters. In both cases (users with/without glasses), the number of clusters is two ($C_{1},C_{2}$) as Figure 1d shows. To ensure that the first position of the clusters is approximately close to the eyes, each one is initialized with the center point of the ellipse. The coordinates of these points are set to half distance of each semi-axis of the ellipse. These initial points guarantee the convergence of the K-means method in few iterations. These initial positions are detailed in Equation (10):

(10) $$\begin{array}{c} C_{1}=\left(cos\left(\beta \right)\times \left(-\frac{R_{min}}{2}\right)-sin\left(\beta \right)\times \frac{R_{max}}{2}\right)+x_{0};sin\left(\beta \right)\times \left(-\frac{R_{min}}{2}\right)+cos\left(\beta \right)\times \frac{R_{max}}{2})+y_{0})\\ C_{2}=\left(cos\left(\beta \right)\times \frac{R_{min}}{2}-sin\left(\beta \right)\times \frac{R_{max}}{2}\right)+x_{0};sin\left(\beta \right)\times \frac{R_{min}}{2}+cos\left(\beta \right)\times \frac{R_{max}}{2})+y_{0}) \end{array}$$

where $C_{1},C_{2}$ are the initial clusters, β is the rotation angle of the ellipse (it will be explained how to compute it later), and $R_{max},R_{min}$ are the distances of major semi-axis and minor semi-axis respectively. The semi-axis distances are calculated using Equation (11):

(11) $$R_{max}=\frac{1}{8}\times \sqrt{2|K|\times \sqrt{B^{2}+{\left(A-C\right)}^{2}}-2\times Q\times \left(A+C\right)}\;R_{min}=\sqrt{R_{max}^{2}-L^{2}}$$

where *K* is the coefficient normalizing factor of the ellipse and *L* is the distance between the center and focal points, as detailed in Equation (12):

(12) $$K=64\times \frac{F\times \left(4\times A\times C-B^{2}\right)-4\times A\times E^{2}+B\times D\times E-C\times D^{2}}{{\left(4\times A\times C-B^{2}\right)}^{2}}\;L=\frac{1}{4}\times \sqrt{\left|K\right|\times \sqrt{B^{2}+{\left(A-C\right)}^{2}}}$$

Regarding the value of β, as Figure 1d shows, it can be obtained with the angle between two director vectors ${\vec{u}}^{\;},{\vec{v}}^{\;}$. The angle β is calculated using Equation (13).

(13) $$\beta =cos^{-1}\left(\frac{{\vec{u}}^{\;}\times {\vec{v}}^{\;}}{\left|\right|{\vec{u}}^{\;}\left||\;|\right|{\vec{v}}^{\;}\left|\right|}\right)$$

where ${\vec{u}}^{\;}$ is the vector formed from the center of the ellipse to the upper point of semi-axis of the ellipse. In addition, $\vec{v}$ is the vector created by the ordinate axis and the central point of the ellipse. Both are calculated using Equation (14).

(14) $$\vec{u}=\left(x_{t}-x_{0},y_{t}-y_{0}\right)\;\vec{v}=\left(0,y_{0}\right)$$

where $x_{t}$ and $y_{t}$ are the coordinates of the upper point of the major semi-axis of the ellipse, and $x_{0}$ and $y_{0}$ are the coordinates of the center point of the ellipse as Figure 1d shows. These points are obtained from Equation (5). Equation (15) is the resulting formula for the upper point.

(15) $$y_{t}=y_{0}+\frac{\sqrt{{\left(2\times B\times D-4\times A\times E\right)}^{2}+4\times \left(4\times A\times C-B^{2}\right)\times \left(D^{2}-4\times A\times F\right)}}{2\times (4\times A\times C-B^{2})}\;x_{t}=\frac{-B\times y_{t}-D}{2\times A}$$

Once β has been found, it is necessary to determine the direction of rotation (clockwise or counterclockwise). The abscissa coordinate of the upper point (used in vector $\vec{u}$) is analyzed. If the abscissa coordinate is lower than the abscissa value of the center point of the ellipse, the rotation is clockwise (β is positive). Otherwise, the rotation is counterclockwise (β is negative).

The fact that the initial clusters are positioned at the places detailed by Equation (10) ensures that the K-means algorithm will converge in fewer iterations. The distance metric to find the clusters in each iteration is the Euclidean distance.

### 3.6. Forehead Segmentation

The detected eye zones (or glasses clusters, accordingly) from the previous step are an indicator that the forehead is close to those points. Hence, a new point is computed which is in the middle of the segment that connects both previously detected points ($M_{p}\left(x_{m},y_{m}\right)$). However, the middle point may still be too close to the eyes or the glasses. To solve this problem, 20% of the length from the calculated middle point and the upper point of the semi-axis of the ellipse is subtracted.

This percentage has been empirically selected minimizing the average error between a ground truth of the forehead and the segmented image obtained in this step, in all the analyzed cases. Moreover, this percentage has been proved to work well on other proposals [21].

Once the new mid-point has been calculated, the forehead region is the area confined by a line $R_{2}$ parallel to $R_{1}$, which is passing through ($Subtracted\_M_{p}$), and the ellipse as Figure 1d shows.

To cut again the ellipse, it is performed setting all the points which satisfy the condition $y_{p}-\left(m\times \left(x_{p}-x_{sm}\right)+y_{sm}\right)<0$ to 0. The result of the forehead region is shown in Figure 1g.

### 3.7. Average Forehead Temperature Extraction

The last step of the proposed method uses the detected forehead region (*forehead_mask*) in the previous step and the raw data obtained by the sensor (*raw_frame*) as Algorithm 1 shows. Hence, the temperature of the forehead corresponds with the average of the non-zero values of the mask applied to the *raw_frame*, as Equation (16) shows.

(16) $$\bar{F_{t}}=\frac{\sum_{i=0}^{n}\sum_{j=0}^{m}P\;\left(x_{i},y_{j}\right)\times \frac{P_{mask}\;\left(x_{i},y_{j}\right)}{255}}{w}$$

where $\bar{F_{t}}$ represents the average temperature of the forehead, *n* and *m* are the number of rows and columns, respectively (being n×m≠0). $P\;\left(x_{i},y_{j}\right)$ is a point of *raw_frame*, and $P_{mask}\;\left(x_{i},y_{j}\right)$ is a point in *forehead_mask*. The amount of non-zero points in *forehead_mask* is represented by *w*.

## 4. Experimental Protocol

To carry out the present study, recordings have been made with the following elements:

- Thermal camera (*Optris PI 160*). Frame rate: 30 Hz; Resolution: 140×120 pixels; Spectral range: 7.5μ−13μ; Field of view (FOV): $6^{\circ }\times 5^{\circ }$; Noise equivalent temperature difference (NETD): 80 mK; Emissivity acquisition value: 0.98.
- Single-Board Computer (*Raspberry Pi 3 model B*): ARM Quad core Cortex-A53 1.2 GHz processor; 1 GB LPDDR2 900 MHz RAM.

Nevertheless, the developed method may work with different devices with similar features, as no special features from those elements are used.

In addition to the proposed method, this paper provides a new dataset of thermal image faces, which is freely accessible in [30].

The images in the database were obtained using the aforementioned devices. The database has more than 18,000 images extracted from different videos recorded to five different users. The users were placed at two meters from the sensor. They should be 30 minutes before the experiment in a still room with a temperature set to 25 °C.

Users were asked to rotate and to flex the neck in different positions:

- Neck flexion: Up to $30^{\circ }$.
- Neck extension: Up to $20^{\circ }$.
- Right lateral neck flexion: Up to $50^{\circ }$.
- Left lateral neck flexion: Up to $50^{\circ }$.
- Right lateral neck rotation: Up to $60^{\circ }$.
- Left lateral neck rotation: Up to $60^{\circ }$.

To control the rotation angles, four different objects were placed at certain angles from the chair where the users sat. This allowed all rotations to be performed except for the side flexes. For the lateral flexions, behind the user, there was a uniform background of painted paper with the limits of the flexions to be performed. Once the recording started, they were asked to turn their heads until they looked directly at the indicated object. In addition, in the case of the side flexes, they were asked to stop when they reached the limit.

The dataset contains: users with or without glasses, users with different facial elements, such as beard, moustache, or none. Moreover, some users had a mask which covers most of their faces, which modifies substantially the morphology of their faces. This feature is hard to find in most thermal face datasets.

All subjects gave their informed consent before they participated in the experiment and allowed the use of their images for researching purposes.

## 5. Results

In this section, the main results and metrics used to test the proper functionality of the proposed method are shown.

**Figure 2:**
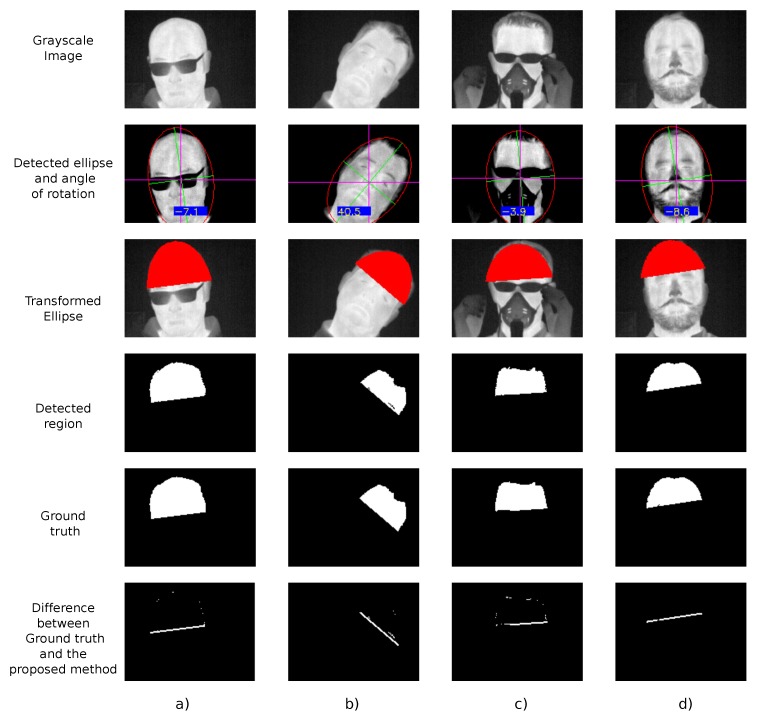
Result of the proposed method for users (**a**), (**b**), (**c**), and (**d**). Where grayscale image, detected ellipse with its angle of rotation, transformed ellipse, detected region of forehead, ground truth of the forehead and the difference between ground truth and proposed segmentation are shown by rows.

The results of the proposed method applied to four different users are shown in Figure 2. Figure 2a shows the main challenge of the forehead detection when a user wears glasses and the neck is slightly rotated and flexed. A user with a lateral neck rotation and not wearing glasses is shown in Figure 2b. A user wearing elements that hide the morphology of his face (a breathing mask and glasses) and other external elements (hands) appear in the image shown in Figure 2c. Finally, Figure 2d shows a user with a moustache and beard which hides the morphology of the face. These four cases show many of the challenges that cannot be resolved by the works cited in Section 2.

Figure 2 also shows other steps of the algorithm such as: the grayscale image, the ellipse part that has the forehead region, the detected forehead region extracted from that ellipse, and the manually selected ground truth of the forehead region.

To test the accuracy of the proposed method compared with the manual segmentation (ground truth), the coefficient of Jaccard, also known as Intersection over Union [31], has been used. The results of the Jaccard coefficient for the cases shown in Figure 2 are detailed in Table 1.

Since the provided data set is quite large, a subset of 1000 images has been selected. For this subset, manual segmentation of the forehead has been carried out to obtain the *ground truth*, to make comparisons with the results provided by the proposed method.

Hence, the last row of Table 1 shows the results of applying the Jaccard coefficient to the aforementioned subset of 1000 images.

The execution times of each step of the proposed method are shown in Table 2, where the time is expressed in milliseconds. Moreover, the total time taken by the proposed method is detailed in the last row of Table 2. These times were obtained using the single-board computer detailed in Section 4. Since the eyes detection and glasses detection steps cannot be given at the same time, the most time-consuming step (users with glasses) has been taken for the total time consumption of Table 2. In this case, the average time of each step was obtained using the complete dataset with 18,000 images.

## 6. Discussion

Analyzing the results of the proposed method as Figure 2 shows, the forehead region is correctly segmented for all users.

For user (a), who wears glasses as Figure 2 shows, his head is slightly rotated (approximately $7^{\circ }$ counterclockwise). In this case, the proposed method can find the lenses of the glasses of the rotated head without problems. It can be observed that the clothes that appear in the grayscale images were removed in the thresholding step and the lenses are emphasized. As the detected region and ground truth images show in that figure, the precision of the proposed method is high. Only a slight line and few points were obtained in the difference of the images because the detected region is a bit smaller than the manual segmentation. This fact is can be observed numerically in Table 1 obtaining a similarity of approximately 94%.

A large clockwise flexion of the neck is shown for user (b) in Figure 2b. In this case, as can be observed with the grayscale image and the thresholded image, not only the clothes were removed, but the hair and the ears too. In this case, as shown in the detected ellipse subimage, not all the points are covered. However, that is not a problem since the whole face is included in the ellipse and the angle of flexion is correctly calculated. The similarity of the proposed method with the manual segmentation is slightly lower (92%) than for user (a). This is because the 20% crop taken in Section 3.6 is small and should be a bit larger than the selected percentage.

The last two users (c) and (d) show the main challenges of the forehead segmentation in Figure 2c,d. In both cases, the morphology of the face is significantly changed. In the case of user (c), he is wearing glasses along with a breathing mask that hide a large part of the face. Moreover, in the selected frame, the user needed to adjust his glasses and his hands appear in the image. As the thresholding step shows, the hands are completely removed from the image and the lenses of the glasses and the breathing mask are emphasized. The detected rotation angle by the method, which is used to initialize K-means clusters, is counterclockwise when the face is aligned with the Cartesian axes (purple axes). This slight rotation does not affect the accuracy of the method (approximately 95% of similarity with the ground truth image), as shown in Table 1.

User (d) is a clear example of the natural morphology modification of the face in contrast to the previous user (c) in which the glasses and breathing mask are external elements. The flexion angle value is clockwise, and the proposed method can be calculated without any difficulties. In this case, as in the rest of the users, the differences in the similarity (below 8%) as happened in the case of user (b) are due to the percentage applied in Section 3.6.

Regarding the accuracy of the forehead segmentation for the subset of 1000 images, as the last line in Table 1 shows, the Jaccard coefficient is over 90% of similarity. This decrease of the similitude is due to those cases when the rotation angle is extremely large or close to the detailed angles in Section 4. Moreover, the selected percentage in Section 3.6 decreases slightly the Jaccard coefficient. However, this value has been proved in the subsect of 1000 images and visually in the full dataset to work well, providing results quite similar to the ground truth.

Finally, Table 2 shows that the processing time for forehead segmentation and temperature measurement is under 12 milliseconds, this is equivalent to 83 frames per second. Thus, real-time processing is achieved. Moreover, this table shows that the step which spends more time corresponds with the eye (or glasses) detection, both based on the K-means algorithm. This large processing time in K-means is due to the iterative approach of the algorithm.

Once the experiments have been analyzed, as was shown in this section, the proposed method is simple and robust. However, the resolution as the distance of the camera plays an important key factor to ensure that enough features will be acquired. It is interesting to remark that thermal cameras acquire thermal radiation emission. In this case, the emissivity of the object must be known and in the unusual case that the background provides the same thermal radiation emission as the skin of a person, thermal cameras are not enough for the extraction of the forehead region and it must be accompanied by other types of sensors as RGB [15]. Although this could solve this limitation, it will have an impact on the performance since the invested time to compute RGB images could be higher than a single 8-bit channel image as is the case of thermal images.

## 7. Conclusions

This work proposes a simple but novel method to segment the forehead of a person using a single thermal camera. This method fills one of the gaps in automatic methods for forehead segmentation and temperature extraction.

The provided method is automatic, with the only required user interaction at the beginning to specify whether the user wears glasses or not. After that, all the steps of the algorithm are automatic without requiring any other user interaction. Moreover, the method is invariant to the morphology of the face when it has facial hair, moustache, respirators, and even strange agents as shown by experiments conducted in which a user brings his hands close to the face.

An advantage of the proposed method is that it can work within real-time constraints, in embedded systems with low resources, as ARM CPU-based single-board computers. In fact, with the tested devices, the method could complete the entire processing at a rate of 83 frames per second (a value twice as large as the frame rate with which the camera acquires images). This feature makes this method ideal for working on real-time systems, monitoring systems, or even to work in one of the related works [2,10,16] analyzed in Section 2.

The Intersection over the Union (IoU), also known as Jaccard coefficient, has been selected to measure the accuracy of the proposed method in the segmentation of the forehead. The method provides an IoU value up to 0.9 (90%) of similarity of the segmentation compared with a manually generated ground truth for a subset of 1000 images.

Another contribution of this work is that it provides a dataset of thermal face images. This dataset provides images of faces with different rotations and neck flexions, which is hard to find feature in any thermal face dataset. In addition to the rotations, it includes cases with glasses, medical breathing masks, beard, whiskers, and various combined elements that modify or hide the morphology of the face. 

## Figures and Tables

**Table 1:** Accuracy of forehead segmentation.

Case	Jaccard Coefficient
User a)	0.9396
User b)	0.9151
User c)	0.9449
User d)	0.9247
Subset of 1000 images	0.9041

**Table 2:** Average time using a Raspberry Pi 3 model B (Single-Board Computer).

Step	Time in Milliseconds
Raw_data_transformation	0.73
threshoding_image	0.92
ellipse_calculation	2.76
ellipse_transformation	1.67
eyes_detection	4.38
glasses_detection	4.86
forehead_segmentation	1.02
forehead_temperature_extraction	4.68 × $10^{-4}$
Total time consumption	11.96

## Abbreviations

The following abbreviations are used in this manuscript:

RGB	Red-Green-Blue channels of an image or type of camera.
HCI	Human-Computer Interaction.
CPU	Central Processor Unit.
FOV	Field of view.
NETD	Noise Equivalent Temperature Difference.
GHz	Gigahertz.
GB	Gigabyte.
mK	milliKelvins.
MHz	Megahertz.
IoU	Intersection over the Union.
